# Case Report: Supernormal Vascular Aging in Leningrad Siege Survivors

**DOI:** 10.3389/fcvm.2022.843439

**Published:** 2022-05-23

**Authors:** Oxana Rotar, Maria Boyarinova, Ekaterina Moguchaya, Kristina Tolkunova, Nikita Kolosov, Valeriia Rezapova, Olga Freylikhman, Dmitrii Usoltsev, Olesya Melnik, Alexey Sergushichev, Vladislav Solntsev, Anna Kostareva, Elena Dubinina, Trudy Voortman, Christine Stevens, Mark J. Daly, Alexandra Konradi, Evgeny Shlyakhto, Mykyta Artomov

**Affiliations:** ^1^Almazov National Medical Research Centre, Saint Petersburg, Russia; ^2^ITMO University, Saint Petersburg, Russia; ^3^Program in Medical and Population Genetics, Broad Institute, Cambridge, MA, United States; ^4^Herzen State Pedagogical University of Russia, Saint Petersburg, Russia; ^5^V.M. Bekhterev National Research Medical Center for Psychiatry and Neurology, Saint Petersburg, Russia; ^6^Erasmus Medical Center, Rotterdam, Netherlands; ^7^Division of Nutrition and Health, Wageningen University, Wageningen, Netherlands; ^8^Analytic and Translational Genetics Unit, Massachusetts General Hospital, Boston, MA, United States; ^9^Institute for Molecular Medicine Finland (FIMM), Helsinki, Finland

**Keywords:** supernormal vascular aging, starvation, aging, deep phenotyping, pulse wave velocity (PWV)

## Abstract

Age-related changes in the vascular system play an important role in the biological age and lifespan of a person and maybe affected from an early age onward. One of the indicators of changes in the vascular system is arterial wall stiffness and its main measure, i.e., carotid-femoral pulse wave velocity (cfPWV). We examined arterial wall stiffness in a sample of 305 Leningrad Siege survivors to assess how hunger and stressful conditions during fetal development and early childhood affected the state of the cardiovascular system at a later age and what factors may neutralize the negative impact sustained in early childhood. Here, we presented an evaluation of two unique patients with supernormal vascular aging (SUPERNOVA) phenotype from this cohort and described the details of congruence between hereditary resistance and practiced lifestyle yielding slower biological aging rate.

## Introduction

The progress of modern medicine contributes to an increase in life expectancy, which leads to a growth in the fraction of aging individuals in the overall population. Therefore, finding actionable health parameters to focus the healthcare system on delaying age-related health deterioration is in great demand.

Various studies emphasize that chronological age and biological age (determined by functional changes in the body) can differ significantly. The concept of biological age is increasingly being used in aging research to determine the true age and rate of aging of an organism. Biological age is a concept that considers the non-uniform character of the aging process in different people, resulting in differences in expected lifespan and variations in health (1). The markers of biological age described to date range from phenotypic and functional indicators to molecular biomarkers.

Arterial wall stiffness is one of the structural indicators of biological age (2). Carotid-femoral pulse wave velocity (cfPWV) is one of the main instruments for assessing arterial stiffness and estimating vascular age. Arterial stiffness is determined by a decrease in the elastin/collagen ratio in the vascular wall, the formation of elastin crosslinks, inflammation caused by reactive oxygen species, calcification, stiffness of the vascular smooth muscle cells themselves, and endothelial dysfunction. Arterial stiffness is thought to be a heritable phenotype, yet due to the uncommon usage of this phenotype in genetic studies, only few DNA loci were associated with it to date (3).

Based on the comparison of vascular age and chronological age, several concepts of vascular aging have been identified. Early vascular aging (EVA), a concept first introduced in 2008, shows premature changes in the structure and function of arteries, reflecting arterial stiffness corresponding to an older chronological age. Normal vascular aging (NVA) has been/is used for indicators of arterial stiffness that are consistent with the chronological age. The concept of a normal “young” arterial elasticity in spite of a medium-high risk factor burden, supernormal vascular aging (SUPERNOVA), was introduced and is actively investigated to understand biological drivers of such an unusual health sustainability (4).

The study of SUPERNOVA as a protective phenotype is of great interest for the prevention of vascular aging, hence protecting against adverse cardiovascular events. The SUPERNOVA phenotype is hypothesized to be a property of the arterial wall to demonstrate different sensitivities or abilities to recover from exposure to various types of damaging factors (5).

Various factors associated with vascular aging can be distinguished. Among them are non-modifiable factors, such as sex and age, and modifiable ones, such as blood pressure (BP), diabetes mellitus (DM), dyslipidemia, and smoking (6, 7).

Experiments have shown that restriction of caloric intake is also associated with a decrease in aging pace (8, 9). Caloric restriction initiates a number of changes in the human body, e.g., improvement of endothelial function, which was shown to slow down vascular aging (10, 11).

Several studies of the relationship between long-term caloric restriction and longevity in animal models have been published to date (12). However, human research on caloric restriction is rather difficult and limited. A unique population for studying the effects of a significant reduction in caloric intake at an early age on the state of the cardiovascular system in the long-term period of life is a sample of residents of besieged Leningrad. The Leningrad Siege during World War II lasted from September 1941 until January 1944 and led to a significant decrease in the amount of food consumed by the city's population. In addition to a decrease in macronutrients and total caloric intake, there was a deficiency of the most important micronutrients involved in the formation and maintenance of vital processes in the body. Such conditions had an impact on the health of individuals exposed in early childhood or *in utero*.

Within the concept of early age programming, it has been demonstrated that the conditions of fetal development, low birth weight, and quality of nutrition at an early age are associated with a risk of cardiovascular diseases, DM, and metabolic syndrome at a later age (13). However, it is feasible that protective factors that arose in the early period of life can neutralize the negative effect of hunger and stress, potentially leading to health benefits later in life.

In this study, we present the results of the examination of two female patients with SUPERNOVA, who survived the Leningrad Siege at different ages, namely, intrauterine/early postnatal age, and the age of 1–3 years, and who participated in the Leningrad Siege survivors study (*n* = 305) (14, 15). We observed a cooperative role of socioeconomic, behavioral, and genetic factors associated with a unique phenotype observed in these patients.

## Materials and Methods

Leningrad Siege survivors (*n* = 305) (14, 15) were invited for an ambulatory visit to the Almazov National Medical Research of the Russian Federation Ministry of Health in 2009–2011, and the follow-up visit took place in 2013–2014. The study was approved by the Local Ethical Committee of the Almazov National Medical Research Center (extracted from protocol no. 243 dated November 12, 2012).

All subjects signed informed consent and were interviewed according to the questionnaire, which includes information about concomitant diseases, therapy, life situations and health conditions during the Siege, and the duration of stay in besieged Leningrad. The prevalence of cardiovascular diseases was recorded based on the data collected during the examination and the doctor's conclusion based on the results of laboratory and instrumental examinations.

Various instrumental studies, assessment of psychological and behavioral factors, and other tests were performed (detailed information on the phenotyping process is available in Supplementary Material) (16–18).

The DNA samples (*n* = 262) were genotyped using the GSA Illumina array version 2.0 at the Broad Institute. The Haplotype Reference Consortium data were used as a reference for genotype imputation using Beagle version 4.0 (19).

Polygenic risk scores for various traits were calculated with PLINK 1.9 (20) using a protocol reported by Martin et al. (21). Genome-wide association studies (GWAS) summary statistics from the UK biobank for another 11 phenotypes (i.e., high-density lipoprotein [HDL], BMI, total cholesterol, triglycerides, height, weight, waist and hip circumstances, glucose level, and systolic and diastolic BP) were downloaded from http://www.nealelab.is/uk-biobank and used for polygenic risk calculations. Polygenic risk score for PWV was calculated using summary statistics from the study described by Rode et al. (22). Histogram plots were built in R studio using the *ggplot2* package (23).

## Results

**Figure 1:**
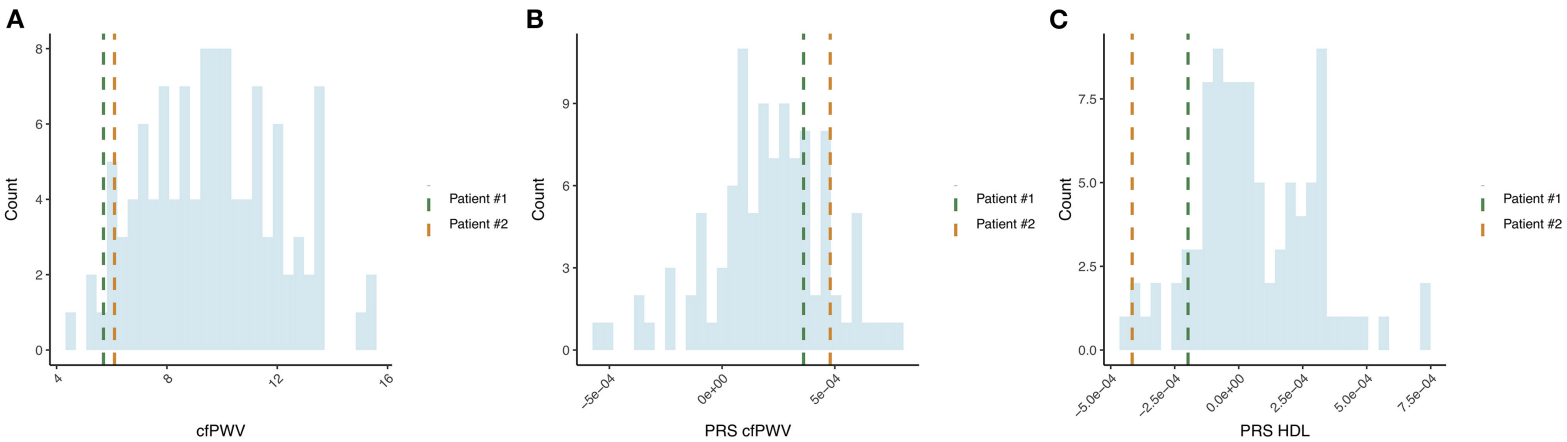
Identification of individuals with SUPERNOVA phenotype and comparison of polygenic scores (PGS) for investigated patients and a control population of the same age and background. **(A)** Pulse wave velocity (PWV) measurements distribution in age-matched cohort of the Leningrad Siege survivors and two selected patients are highlighted; **(B)** PWV PGS, i.e., cfPWV; **(C)** High-density lipoproteins polygenic score.

The original cohort of 305 survivors of the Leningrad Siege was analyzed to identify individuals without a history of antihypertensive and statin medication usage, BMI < 30, no history of hypertension, dyslipidemia, ischemic heart disease, diabetes, and renal dysfunction. The cfPWV was assessed using a SphygmoCor (AtCor, Australia) by applanation tonometry. The following reference values of cfPWV were used for people over 60 years of age (median, 10th and 90th percentiles), namely, 60–69 years, i.e., 9.7 m/s (7.9–13.1 m/s) and 70 years, i.e., 10.6 m/s (8.0–14.6 m/s). Based on these values, we conditionally isolated the phenotype of SUPERNOVA, i.e., the cfPWV value was less than the 10th percentile for this age group. Two patients satisfied all criteria and were selected for the detailed analysis (Patient 1: 7.6 m/s, Patient 2: 5.7 m/s at the second visit, Figure 1A).

In addition, though it was not used as an inclusion criterion, we confirmed younger than expected estimated biological age of the vessels using cardio-ankle vascular index (CAVI) values (Supplementary Tables 1, 2).

### Female Patient 1

The patient was born on September 10, 1940. At the onset of the Leningrad Siege in World War II (September 08, 1941), she was 1 year old. The patient spent the entire Siege period in Leningrad. Lifestyle factors are presented in Supplementary Table 1.

At the time of examination in 2010, the patient was 69 years old. The survey did not reveal any cardiovascular complaints, and there was no indication of a history of arterial hypertension: the measured BP was 115/70 mm Hg, maximum self-reported BP was 130/80 mm Hg, and the patient was not receiving antihypertensive therapy. There were no indications of a history of cardiovascular disease or DM. In 2008, during surgical treatment of cataracts, lens replacement was performed, and the history of chronic cholecystitis was revealed. Gynecological history was unremarkable. According to the patient's complaints, an increase in total cholesterol level of 5.5–6.4 mmol/L was previously detected. The patient was advised to monitor BP and pulse, lipids, and blood glucose levels.

In 2013 (at the age of 73 years), at the time of the next visit, the patient maintained normal BP, and no cardiovascular events were recorded. During the past study period, the lens of the second eye was replaced (2012), autoimmune thyroiditis with moderate hypothyroidism was revealed, and, therefore, levothyroxine sodium was prescribed with the appearance of euthyroidism.

Examination results (2010 and 2013) and patient questionnaire data are shown in Supplementary Table 2.

### Female Patient 2

The patient was born on August 21, 1942, 11 months after the beginning of the Leningrad Siege; the prenatal period fell in 1941, the most difficult year of Siege in terms of food supply availability. The patient was evacuated from Leningrad at the age of 5 months. Lifestyle factors are presented in Supplementary Table 1.

At the time of baseline examination in 2010, the patient was 68 years old. The history of patient, since 2002, revealed mild hypertension with maximum BP of 150/90 mm Hg but the patient had not received antihypertensive therapy. At the age of 13 years, she suffered from acute rheumatic fever. The examination according to echocardiography in 2010 revealed mitral insufficiency of grades 1–2. There were no indications of a history of cardiovascular complications, DM, or other comorbidities. The patient denied a previous increase in blood glucose and cholesterol levels. Gynecological history was unremarkable.

The 2013 follow-up examination was performed at 71 years old. The patient had the same mild hypertension, and no cardiovascular events were recorded. Examination results (2010 and 2013) and patient questionnaire data are shown in Supplementary Table 2.

Analysis of the EPFQ Nutritional Questionnaire showed that the diets of both patients at the follow-ups in 2010 and 2013 were similar and included a moderate amount of simple carbohydrate-rich foods (e.g., pastry), i.e., an average of 3 servings per day; 3 servings of complex carbohydrates per day; a sufficient number of servings of vegetables and fruits (3–4 servings per day); 1–2 servings per day of protein-rich foods (e.g., dairy products, meat, fish, and eggs); and there was no consumption of fast food and there was practically no tertiary processed food (maximum once a week). Low consumption of sugar and sweets was noted.

In addition, we conducted a detailed psychological assessment to identify whether personality factors may contribute to the observed phenotypes (Supplementary Table 3), yet no extreme phenotype was detected using HADS, EQ-5D, SF-36, PLO, or F-SozU 22 scales.

We investigated the inherited susceptibility to maintain healthy vessel stiffness in patients by estimating their individual polygenic scores (PGS). The previously published GWAS for cfPWV was used as a reference (22). To evaluate the population risk profile, we used an age-matched cohort of 103 individuals (67–69 years) of Siege survivors from St. Petersburg. Despite the presentation of SUPERNOVA in the two patients of interest, their PGS for cfPWV was not notable (Figure 1B), suggesting that the observed vessel stiffness could not be fully attributed to inherited risks.

Next, we evaluated PGS for 11 clinical phenotypes that could potentially influence the supernova aging phenotype (Supplementary Figure 1). Interestingly, Patient 1 had high PGS (top 5% of the comparison cohort) for systolic and diastolic BP, yet the observed BP readings were within normal range at both visits. None of the PGS provided a definitive association with the supernova phenotype, except PGS for HDLs. Both patients had PGS for HDL in the lower 5% of the populational distribution obtained from the matched control group (Figure 1C).

Interestingly, HDL measurements for both patients were above the reference values, indicating “normal” level. Previous observations suggest that such interplay between genetically determined and actual measurements could lead to a stronger risk or protection against the study phenotype (24).

## Discussion

Presentation of two clinical cases with SUPERNOVA from the unique population of Leningrad Siege survivors provides insights into the effects of starvation and stress conditions during intrauterine development and early childhood.

We illustrated that in the course of life, a whole set of determinants can form a favorable individual trajectory of cardiovascular health, even despite the existing exposure to extremely unfavorable environmental factors at an early age. As a limitation of our study, we recognized a modest sample size, especially for the genetic component of the study; however, given the unique nature of the cohort study, it is nearly impossible to sufficiently increase the cohort size at this time.

Both patients were not obese, and probably the absence of metabolic syndrome with accompanying insulin resistance and subclinical inflammation is one of the keys to the persistent SUPERNOVA phenomenon in these patients. At the same time, the patients were overweight, and several reports have shown that the presence of overweight has a protective effect in the older age, namely, a lower risk of overall premature mortality and a better prognosis for chronic heart failure (25).

Despite the significant stress factors of the early age period, both patients showed a maintenance of normal reproductive function and the timely onset of menopause that may also be associated with a healthy aging trajectory (26).

Both patients had long-livers in the family. Genetic, environmental, and behavioral factors passed down from generation to generation have been shown to influence the results of aging in offspring. Women whose mother and father lived for 90 years were more likely to achieve healthy aging (27).

Dietary preferences are one of the important modifiable lifestyle factors that can affect the maintenance of the healthy aging phenotype. The study of diet patterns has highlighted some of the key ingredients associated with longevity and improved cardiometabolic and cognitive health (28). The diet of the examined patients did not belong fully to any diet associated with healthy longevity; however, the patients also did not have an unfavorable dietary pattern characteristic of the Western type of diet: no fast-food consumption and low sugar consumption were indicated. There was an adequate intake of fiber (e.g., vegetables and fruits).

Both patients consumed moderate amount of alcohol. In a Rotterdam study of 3,235 participants who aged 61–96 years, a positive effect of drinking moderate doses of alcohol on vascular stiffness was demonstrated, i.e., a decrease in cfPWV (29). Furthermore, both patients had never smoked and had an adequate level of daily physical activity.

Social activity is associated with healthy aging and the retention of the ability to learn. In a study of 1,052 persons (average age of 60.2 years), it was demonstrated that upon adjusting the lifestyle covariates, social support had a positive association with thinking speed and flexibility, and that verbal interactions were connected with verbal learning and memory (30). Female Patient 2, according to psychological examination, had significant personal and social resources that determine a satisfactory quality of life and, therefore, can be associated with healthy aging. Female Patient 1 showed a negative impact of emotional and physical problems on daily activities, as well as low satisfaction with social support, but the results of the psychological evaluation indicated a good cognitive function. Maintaining a high cognitive status is considered one of the key components of healthy aging. In a study of the life expectancy of residents of the Netherlands aged 65 years and above, the authors demonstrated that life expectancy with good cognitive health showed an increase that continued throughout the whole study period (31). Both patients were widows and lived alone. While the death of a husband is clearly a stressful factor, there are paradoxical studies demonstrating the benefits of living alone for the cognitive function of older adults (32).

Healthy longevity can be assumed as a chance occurrence of an ideal congruence between hereditary resistance and practiced lifestyle (33). Presentation of these patients suggests that in certain cases, cumulative effect of favorable lifestyle patterns (behavioral and socioeconomical) and absence of severe genetic risks could be sufficient to mitigate damaging exposures sustained earlier in life.

## Data Availability Statement

The original contributions presented in the study are included in the article/Supplementary Materials, further inquiries can be directed to the corresponding author/s.

## Ethics Statement

The study was approved by the local Ethical Committee of the Almazov National Medical Research Center. Extract from protocol No. 243 dated 12.11.2012 and the Institutional Review Board of Massachusetts General Hospital (IRB #2014P000459). The patients/participants provided their written informed consent to participate in this study. Written informed consent was obtained from the individual(s), and minor(s)' legal guardian/next of kin, for the publication of any potentially identifiable images or data included in this article.

## Author Contributions

OR, MB, AKon, and MA designed and conceived the study. OR, MB, EM, KT, NK, VR, DU, AS, VS, ED, and TV analyzed clinical and genetic data. OF, CS, OM, and AKos collected and processed biospecimen. AKon, ES, MD, and MA acquired funding. OR, MB, VR, and MA wrote the original text. MD, AKon, ES, and MA supervised the study. All authors edited and reviewed the text.

## Funding

OR, MB, EM, KT, NK, VR, OF, OM, AKos, AKon, and ES were supported by the Ministry of Science and Higher Education of the Russian Federation (Agreement No. 075-15-2022-301). CS and MD were supported by Broad Institute SPARK Award. MA was supported by funding from the Aging Biology Foundation.

## Conflict of Interest

MD is a founder of Maze Therapeutics. The remaining authors declare that the research was conducted in the absence of any commercial or financial relationships that could be construed as a potential conflict of interest.

## Publisher's Note

All claims expressed in this article are solely those of the authors and do not necessarily represent those of their affiliated organizations, or those of the publisher, the editors and the reviewers. Any product that may be evaluated in this article, or claim that may be made by its manufacturer, is not guaranteed or endorsed by the publisher.
