# Supplementary material for: Case Report: Supernormal Vascular Aging in Leningrad Siege Survivors
Source: Front Cardiovasc Med. 2022 May 23;9:843439. doi: 10.3389/fcvm.2022.843439 (PMC9170230; doi:10.3389/fcvm.2022.843439)
Supplement: Supplementary file 1 [file Data_Sheet_1.pdf]

A Case Study of Supernormal Vascular Aging in Leningrad Siege Survivors

Oxana Rotar[1], Maria Boyarinova[1], Ekaterina Moguchaya[1], Kristina Tolkunova[1], Nikita Kolosov[1,2,3], Valeriia Rezapova[1,2,3], Olga Freylikhman[1], Dmitrii Usoltsev[1,2,3], Olesya Melnik[1], Alexey Sergushichev[2],Vladislav Solntsev[1], Anna Kostareva[1], Elena Dubinina[4,5], Trudy Voortman[6,7], Christine Stevens[3], Mark J. Daly[3,8,9,*], Alexandra Konradi[1,2], Evgeny Shlyakhto[1] and Mykyta Artomov[1-3,8,9*]

[1] – Almazov National Medical Research Centre, Saint-Petersburg, Russia
[2] – ITMO University, St. Petersburg, Russia
[3] – Broad Institute, Cambridge, USA
[4] - Herzen State Pedagogical University of Russia, Saint Petersburg, Russia
[5] – V.M. Bekhterev National Research Medical Center for Psychiatry and Neurology, Saint Petersburg, Russia
[6] – Erasmus Medical Center, Rotterdam, The Netherlands
[7] – Division of Nutrition and Health, Wageningen University, the Netherlands
[8]– Analytic and Translational Genetics Unit, Massachusetts General Hospital, Boston, USA
[9] – Institute for Molecular Medicine Finland (FIMM), Helsinki, Finland

[*] – correspondence: artomov@broadinstitute.org

The authors declare no conflict of interests

Key words: Supernormal vascular aging, Starvation, Aging, Deep phenotyping, Pulse-wave velocity (PWV).

Phenotyping information 3
Table S1 5
Table S2 6
Table S3………………………………………………………………………….8
Figure S1... 9
References 10

Phenotyping information

Anthropometry was performed on both visits: measurement of height and body weight with the calculation of body mass index (BMI) according to the Quetelet formula, measurement of waist circumference. Blood pressure (BP) and pulse rate were measured using the OMRON device (Japan) on the right hand after 5 minutes of rest in a sitting position three times with the calculation of the average value of the last two measurements. After obtaining the mean values of systolic and diastolic blood pressure (SBP and DBP), pulse pressure (PP) was calculated as the difference between SBP and DBP.

A fasting blood sample was taken with the determination of the lipid profile (total cholesterol, high density lipoproteins, low density lipoproteins, triglycerides), glucose level. At the second visit the creatinine level was additionally determined with the calculation of the glomerular filtration rate (GFR) according to the CKD-EPI formula. The morning urine sample was collected to determine the concentration of albumin and creatinine, followed by the calculation of the albumin-creatinine ratio. The analysis of blood and urine parameters was performed on an Abbot Architect c8000 biochemical analyzer (USA).

At both visits electrocardiogram (ECG) was registered using PADSY computerized complex ("Medset Medizintechnik GmbH", Germany), interpretation was carried out according to a standard protocol.

At the first visit the following was performed:

- carotid ultrasonography: assessment of the morphology of the carotid arteries, detection of signs of atherosclerotic damage and calculation of TIM (thickness of intima-media). We used a Vivid 7 (Samsung, Korea (General Electric, USA), 7 MHz high-resolution transducer.

- echocardiography. The study was carried out on a Vivid 7 (GE, USA), a 3.25 MHz transducer in M-modal, two-dimensional and Doppler modes in standard echocardiographic positions along the short and long axes.At the second visit, the cardio-ankle vascular index (CAVI) and ankle-brachial index (ABI) were determined on the right and left using the VaSera VS-1500 device (FukudaDenshi, Japan).

At both visits, the carotid-femoral pulse wave velocity (PWV) was assessed using a SphygmoCor device (AtCor, Australia) by applanation tonometry. Reference PWV values by age group were taken from the paper "The Reference Values for Arterial Stiffness Collaboration", based on data from a large European study[1]. For people over 60 years of age, the following PWV indicators (median, 10th and 90th percentiles): 60-69 years - 9.7 (7.9-13.1) and -70 years - 10.6 (8.0-14.6). Based on these values, we conditionally isolated the phenotype (or phenomenon) of supernormal vascular aging (SUPERNOVA) - the PWV value less than the 10th percentile for this age group.

Cognitive functioning was assessed by the Mini Mental State Examination (MMSE) – a brief (11 tasks) quantitative screening measure of cognitive status in adults[2,3]. The cut-off point for cognitive impairment was 26 points and lower.

Anxiety and depression were assessed using a Russian validated version of the Hospital Anxiety and Depression Scale (HADS)[4]. Cut-offs for increased level of anxiety and depression were 8 points and higher.

Two general instruments for assessing health-related quality of life were used: EQ-5D (3-level version) and 36-Item Short Form Survey (SF-36).
The sense of meaningfulness and purpose in life was assessed by the Purpose in Life Orientation Test (PLO)[5]. The short version (K-22) of the Social Support Questionnaire F-SozU (Fragebogen zur Sozialen Unterstützung) was used to assess perceived functional social support.

At the second visit, the patients completed the EFPQ (European Food Propensity Questionnaire), which includes 116 questions regarding the consumption of various food and beverage groups.

Patients clinical presentation

Table S1
Lifestyle factors

Parameter	Patient No. 1	Patient No. 2
Breastfeeding after birth	Delivered	Delivered
Living conditions during the blockade	Room in a communal (shared) apartment	Room in a communal (shared) apartment
Number of people in a communal apartment	3	5
Diseases during the blockade	Acute respiratory diseases	Childhood infections<br>Cachexia
Gynecological anamnesis	Pregnancy – 1. Childbirth - 1	Pregnancy – 3. Childbirth – 1, Abortion - 2
Menopause start	47 years	55 years
Education	Higher education	Secondary education
Family status	Widow	Widow
Heredity: mother	Death at 89 years old, was overweight and hypertensive	Death at 79 years old, was overweight.
Heredity: father	Death at 40 years old (during the war), diseases are unknown	Death at 94, diseases are unknown
What part of income is spent on food?	1/3	1/3
Alcohol consumption	Once a month, 3 doses (spirits)	Several times a week ½ dose (wine)
Smoking	Never smoked	Never smoked
Physical activity	Daily walk 60 min.<br>Rare moderate physical activity for 20-30 minutes	Daily walk 60 min.<br>Rare moderate physical activity for 20-30 minutes
Sleep duration, hours	7	8
Sitting time, hours	5	10
Snoring	Snoring - unknown.<br>Sleep apnea - no	Snoring - yes.<br>Sleep apnea – unknown
Pet	No	Has a cat

Table S2

Results of laboratory and instrumental investigation

Parameter	Target value	Patient No. 1		Patient No. 2	
		Visit 1	Visit 2	Visit 1	Visit 2
Date of examination		14.04.2010	20.12.2013	13.04.2010	12.12.2013
Age, years	-	69	73	68	71
Height, cm	-	158	153	154	148
Weight, kg	-	67,0	66,4	58	62
BMI, kg / m²	< 25	26,9	28,2	24,4	28,3
Waist circumference, cm	< 80 (f)	90	89	75	83
SBP sitting mean, mm Hg	< 140	122	110	133	148
DBP sitting mean, mm Hg	< 80	80	68	76	81
SBP standing mean, mm Hg	-	-	115	-	144
DBP standing mean, mm Hg	-	-	78	-	90
PP sitting mean, mm Hg	< 60	42	42	57	67
Pulse, beats / min	≤ 80	64	64	60	65
Hypertension diagnosis	No	No	No	Yes	Yes
Antihypertensive therapy	No	No	No	No	No
Glucose, mmol / l	< 5,6	5,3	4,9	4,9	5,0
TC, mmol / l	≤ 4,9	5,4	5,4	4,8	4,2
HDL, mmol / l	≥ 1,2	1,3	1,1	1,6	1,4
LDL, mmol / l	< 2,6	-	3,8	-	2,5
TG, mmol / l	< 1,7	1,1	0,9	0,58	0,6
Lipid-lowering therapy	No	No	No	No	No
GFR ml/min/1.73 m^2	≥ 60	-	78,3	-	88,9
ACR in a single portion of urine, mg / mmol	< 3,4	-	0,5	-	1,4
Duplex ultrasound of the carotid arteries	IMT <0.9 mm	IMT 1.1 mm bilaterally	-	IMT 1.1 mm bilaterally	-
LVMI, g / m^2	≤ 95 (f)	130	-	83	-
LVDd/LVSd, mm	≤ 52,2/34,8 (f)	44/27	-	44/28	-
Zone of left ventricle myocardial contractility disorder	No	No	-	No	-
EF, according to Simpson, %	> 55	71	-	72	-

LV diastolic dysfunction	No	No	-	No	-
ECG		Sinus rhythm, HR 65 bpm	Sinus rhythm, HR 57 bpm	Sinus rhythm, HR 61 bpm	Sinus rhythm, HR 81 bpm
**PWV, m / s**	**≤ 10**	**6,1**	**7,6**	**no data**	**5,7**
CAVI right	< 9,0	-	7,7	-	8,4
CAVI left	< 9,0	-	8,1	-	8,3
Estimated age of arteries (according to CAVI)			65-69 years		65-69 years
ABI right	> 0,9	-	1,15	-	1,05
ABI left	> 0,9	-	1,03	-	1,09
MMSE, points	29-30	28	29	25	26

BMI - body mass index, f – female, SBP - systolic blood pressure, DBP - diastolic blood pressure, PP - pulse pressure, TC - total cholesterol, LDL - low density lipoproteins, HDL - high density lipoproteins, TG - triglycerides, GFR - glomerular filtration rate, ACR - albumin / creatinine ratio, IMC - Intima-media complex, LVMI - left ventricular myocardial mass index, LVDd/ LVSd - left ventricular end-diastolic dimension / left ventricular end-systolic dimension, EF - ejection fraction, ECG – electrocardiogram, HR – heart rate, PWV - pulse wave velocity, CAVI - cardio-ankle vascular index, ABI - ankle-brachial index, MMSE - Mini-Mental State Examination

Table S3

Results of psychological assessment

Instrument	Subscales	Patient 1	Patient 2
HADS	Anxiety	11	11
	Depression	2	7
EQ-5D		11122	21112
SF-36	Physical Activity	60	40
	Role Physical	25	50
	Bodily Pain	51	62
	General Health	62	50
	Vitality	55	55
	Social Functioning	75	50
	Mental Health	0	100
	Role Emotional	52	76
PLO	life goals,	31	28
	life process,	26	36
	life efficiency	27	22
	locus of control – Self	21	17
	locus of control – Life	25	36
	total score	97	104
F-SozU 22	emotional support,	40	45
	instrumental support,	18	20
	social integration	30	29
	satisfaction with social support	2	6
	total score of social support	90	100

HADS – Hospital Anxiety and Depression Scale, SF-36 - The 36-Item Short Form Survey, PLO - Purpose in Life Orientation Test.

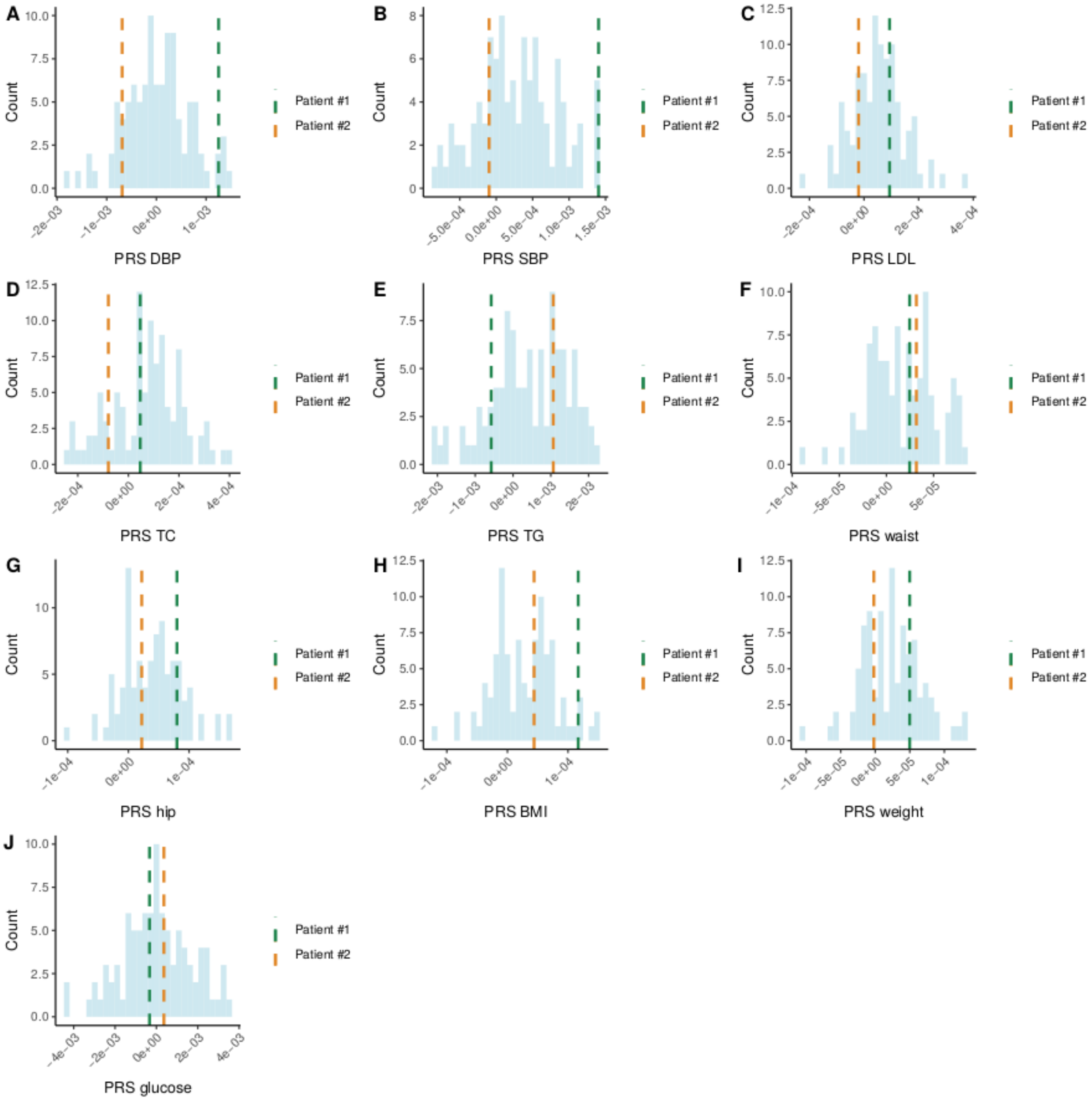

Figure S1.

Polygenic risk score distribution for (A) diastolic blood pressure (DBP), (B) systolic blood pressure (SBP), (C) light-density protein (LDL), (D) total cholesterol (TC) level, (E) triglycerides (TG) level, (F) waist circumference, (G) hip circumference, (H) body mass index (BMI), (I) weight, (J) glucose level.

References

1. Mattace-Raso, F. U. S. *et al.* Determinants of pulse wave velocity in healthy people and in the presence of cardiovascular risk factors: 'establishing normal and reference values'. *Eur. Heart J.***31**, 2338–2350 (2010).

2. Monroe, T. & Carter, M. Using the Folstein Mini Mental State Exam (MMSE) to explore methodological issues in cognitive aging research. *Eur. J. Ageing***9**, 265–274 (2012).

3. Folstein, M. F., Folstein, S. E. & McHugh, P. R. 'Mini-mental state'. A practical method for grading the cognitive state of patients for the clinician. *J. Psychiatr. Res.***12**, 189–198 (1975).

4. Zigmond, A. S. & Snaith, R. P. The hospital anxiety and depression scale. *Acta Psychiatr. Scand.***67**, 361–370 (1983).

5. D.A. Leontiev. Test Smyslozhiznennykh orientatsii (Life Meaning Orientations Test). *Smysl* (1992).
